# Book Reviews

**Published:** 1815-08

**Authors:** 


### CRITICAL ANALYSIS

### *OF RECENT PUBLICATIONS*

IN THE

### DIFFERENT BRANCHES OF PHYSIC, SURGERY, AND MEDICAL PHILOSOPHY.

---

*A Treatise on the Diseases of Arteries and Veins, containing the Pathology and Treatment of Aneurisms and Wounded Arteries.* By JOSEPH HODGSON, Member of the Royal College of Surgeons in London. 8vo. with Plates. pp. 604. Underwood, London.

THIS work, as appears by the preface, is a regular arrangement of two sets of papers presented to the London College of Surgeons. The one a dissertation which obtained the Jacksonian prize; the other, a collection of cases illustrating some parts of the subject which had been imperfectly considered in the dissertation. The whole makes 600 pages of fair modern printing besides engravings, which, being on a larger scale, are contained in a separate volume.

In considering a work of such magnitude and importance, we shall attend with minuteness to some particular chapters, and offer a general view of the remainder. By these means, we can enable the author to speak for himself whenever we notice his opinions, a method which we always prefer as the only fair mode of criticism. We cannot but condemn an affectation lately introduced by reviewers, of endeavouring to give all their articles the appearance of original essays. By this artful contrivance, a hackney-writer borrows as much as he pleases from the work before him without acknowledging the source, and, when he censures his author, often palms opinions upon him not to be met with in his writings. In other words, by appropriating all that is good to himself, and imputing whatever absurdity he thinks proper to his author, it is easy to make himself appear to advantage, and to dress his author like a scaramouch. Thus, the critic appears as the principal performer in the play, and the author is introduced as a butt in the afterpiece.

The first part is on *Diseases of Arteries in General.* In the introduction to this division, Mr. H. remarks, "that arteries are composed of [we suspect he means have as part of their composition] blood vessels, nerves, and absorbents, which render them liable to the same morbid alterations, and endow them with the same powers of reparation as soft parts in general."

"Thus, (continues he,) the coats of arteries inflame and pass through all the stages of adhesion, suppuration, or gangrene, in the same manner as the skin, a gland, or a muscle. But these elementary textures are in arteries so modified or arranged, as to form structures possessing peculiar properties, essential to the functions of the vessel. From this circumstance, arteries are liable to particular diseases arising from the peculiarities of their formation and functions. Thus, certain depositions and alterations take place in their coats, which we do not meet with in other structures; and their cavities undergo changes, which depend upon the actions of the vessels, and their subserviency to other organs.

"With this view of the subject, I shall first consider those morbid changes which arteries undergo in common with soft parts in general; namely, inflammation and its consequences—the effusion of lymph and adhesion, suppuration, ulceration, and gangrene; and, secondly, those changes which arise from peculiarities in the structure of arteries, as calcareous depositions, and various morbid alterations in their coats, and preternatural dilatation of their cavities; and I shall then consider the subject of aneurism, as arising from one or other of these previous changes, or from accidental violence."

The first section is *on Inflammation of the Coats of Arteries.* The grand object of this section is to shew that "though the internal coat of an artery differs essentially from every other membrane in the body—still, in one circumstance, it bears a striking analogy to serous membrane, namely, in its tendency to assume the adhesive inflammation." We cannot object to these positions, but we sincerely wish more attention were paid to the imperfect knowledge of those numerous readers who, we trust, will be enlightened by the industry, and we ought to add, by the genius, of this writer. For these, a short chapter on the Structure of Arteries; another, on the Serous Membranes, or on Membranes in General; and, another, on the Adhesive Inflammation, would have been very useful, —very little more than definitions, with a reference to the various writers, might have been sufficient. It is to be remarked, that these terms are not only adopted from different authors, but even from authors of different nations, speaking different languages, and, perhaps, not sufficiently acquainted with each other to admit of their immediate coalescence without some introduction. Some of our readers, who left school when we did, and have since been engaged in the fatigues of provincial practice, may not be aware that of late it has been the custom, we presume for convenience, to call those membranes which line cavities *serous membranes*, because, under disease, serum is effused from them.

them. But this is neither their constant, nor even their most common, mode of action under inflammation, nor is it the kind of action which Mr. Hodgson wishes to describe in inflamed arteries, and in the manner in which he compares them to such membranes.

"Although the internal coat of an artery differs essentially from every other membrane in the body, in its extreme tenuity and elasticity, the facility with which it is ruptured, and the peculiar unctuous appearance of its internal surface, still in one circumstance it bears a striking analogy to serous membranes, namely, in its tendency to assume the adhesive inflammation. This property is in blood vessels, as in all organs, the first agent of reparation in injuries from accident or disease, and is surprisingly manifested in the processes which effect the cure of a wounded or divided artery. The inflammation which is excited by the injury, produces an effusion of lymph which seals the extremity of the divided vessel, and, extending to its internal coat, becomes the basis of adhesion and final obliteration. Punctured arteries also are united by the same adhesive process that repairs wounds in general. If irritation be excited in the coats of an artery by pressure, the adhesive inflammation is the consequence; an effusion of lymph takes place into the cellular membrane that connects the coats of the vessel, and into its cavity; its sides coalesce, and it is rendered impervious. This effect of pressure upon an artery is illustrated by the obliteration of blood vessels compressed by large tumours, and by that cure which aneurisms occasionally undergo from the pressure of the sac upon the superior or inferior portion of the artery. The same adhesive process frequently prevents hæmorrhage where abscesses or extensive ulcerations exist in the neighbourhood of large blood vessels; for the inflammation which precedes the suppuration has in such cases produced an effusion of lymph between the coats and into the cavity of the artery, whereby it is obliterated. Thus, in cases of vomica, where the substance of the lungs is much consumed, hæmorrhage is prevented by the closure of the branches of the pulmonary and bronchial arteries by the adhesive inflammation.

"The most perfect demonstration, however, of the effect of acute inflammation upon the internal coat of an artery, is probably to be met with in those cases where the disease appears to have extended to the vessel from contiguous parts. The following case of extensive inflammation of the thoracic viscera, in which the disease existed also in the internal coat of the aorta, and had produced an effusion of lymph into the cavity of that vessel, was communicated to me by my friend, Dr. Farre."

We wish our readers to attend carefully to the above passage, and to our remarks, that by thus clearing the ground *in limine*, we may meet with fewer difficulties in our progress. The analogy between the internal coats of an artery and serous membranes, is "in their tendency to assume ad-

 hesive hesive inflammation," and this adhesive inflammation is afterwards described as "the effusion of lymph which seals the extremities of divided vessels." What then are these serous membranes; and, if they have any claim to such a term, what analogy have they with parts which effuse lymph? The writer, we are aware, will refer us to Bichât, and to some of his followers in England, but these works are not in every hand; and, if Mr. Hunter's language is adopted in the term "adhesive inflammation," why are not these membranes called after him, the membranes which line cavities and the cellular membrane, terms which cannot lead into error or confuse. Nor are we satisfied with the mere expression, that "the inflammation produces an effusion of lymph;" mere effusion would be very inadequate to the purpose of *sealing the divided extremities*, were not this lymph effused in a particular form, that is, were it not immediately separated from the serum and the red particles, and so organized by the formation of vessels communicating with the parts to which it adheres as to maintain its life and the situation it has assumed for the important purposes ascribed to it by the author in the above quotation.

Let us now attend to Dr. Farre's case, and the illustrations added by Mr. Hodgson.

"A man (we are told), who had recently returned from Jamaica, where he had been severely afflicted with dysentery, was attacked with violent pneumonia, which destroyed him in the course of five days. The cavities of the pleura were found to contain much lymph and serum. The pericardium was covered with lymph. The cells of the lungs were filled with bloody serum and the bronchia were highly inflamed. All the thoracic viscera exhibited the effects of the highest degree of acute inflammation which had extended also to the aorta, the internal coat of which was of a deep red colour, and a considerable effusion of lymph had taken place into its cavity. The effused lymph was very intimately connected with the internal coat of the vessel, and a plug of it had extended into the left subclavian artery, and nearly obliterated the cavity of that vessel.

"This case exhibits the effects of acute inflammation upon the internal coat of an artery, and shews the tendency which that membrane possesses to assume the adhesive inflammation. A similar state of the great blood vessels is occasionally met with in violent inflammations of the thoracic viscera. I have seen it in three cases of carditis, pneumonia, and bronchitis, but in none had the effusion proceeded to so great an extent as in the instance above related. In one case, the aorta was throughout of a deep scarlet colour; the posterior mediastinum was gorged with serum, and, a little above the semilunar valves, the cellular membrane that connects the coats of the aorta was distended with lymph. This condition of the great arteries has been but little attended to by pathological pathological writers. Morgagni and Boerhaave indeed mention its existence, and impute to it some of the symptoms of suffocation and oppression of the heart, that have been noticed in many thoracic diseases. Portal also remarked it in a young man who died a few days after the repulsion of an acute eruption. The thoracic aorta was very red, swollen, and tender, and its internal coat near the diaphragm was particularly puffed and softened. A similar effusion of lymph sometimes takes place in the neighbourhood of ulcerations and calcareous depositions in arteries. I have seen an ulcerated and thickened artery, where a plug of lymph, evidently effused by inflammation, filled the cavity of the common iliac.

"Lymph, which is thus effused into arteries, sometimes becomes the matrix of vessels, and granulations or fungous growths are occasionally the consequences. Granulations are not unfrequently met with at the origin of the aorta, particularly upon the semilunar valves, and also in the cavities of the heart, the lining membrane of which is continuous with that of the arteries, and appears to possess the same properties.

"The inflammation which is excited in an artery by the application of a ligature, is sometimes propagated to a considerable extent along the vessel. I have seen the inflammation of the internal coat extend even to the heart after the ligature of the femoral artery in amputation, and I have known a similar effect produced by the application of a ligature for the cure of an aneurism in the upper extremity. Mr. Cline and Mr. Abernethy have also observed this circumstance after the ligature of the femoral artery for the cure of aneurisms; and it has been remarked in the hypogastric arteries, after the ligature of the umbilical cord."

Here every part was violently inflamed, and the blood partook of the same increased action, the only effect of which is its coagulation with greater or less rapidity and firmness. That the blood often coagulates in the internal coat of an artery cannot be questioned, but this should not be called the adhesive inflammation, as adhesions are not always formed, and sometimes the coagula are formed without any apparent inflammation in the vessel. In cases of extensive mortification, the blood will coagulate in the vessels leading to the heart; under extensive suppurations the same will take place, and, in the article of death, it is well known, that coagula are often formed in the heart, or the larger vessels, preserving the exact figure of the cavities. Even the red appearance of an artery is, as Mr. Hodgson remarks, a very uncertain test of inflammation.

"The internal coat is of a deep scarlet colour, sometimes throughout the whole extent of the system; and on other occasions this appearance is to be observed only in circumscribed patches. It is attended with no deposition of lymph, or thickening of the vessel; and, if the internal coat be removed, the middle generally presents presents its natural appearance; whereas, in the cases of acute inflammation which I have examined, the middle coat has always exhibited a preternatural degree of vascularity. This red appearance of the internal surface of an artery is often observed in the vicinity of coagula, and in those instances may probably be the effect of transudation after death. But I have very frequently remarked it where no coagulum has been found in the vessel, and it is generally to be observed in arteries that have been long exposed to the air in the dissecting room. I have, however, seen it in subjects that have been inspected a very few hours after death, and cannot, therefore, regard it as produced merely by exposure to the air. Whether it is to be considered as a change which takes place after death, or as a morbid appearance, I am unable to determine. Corvisart says, that it has been regarded as the cause of a peculiar fever, and that Dr. Frank had observed it in nineteen instances of that disease."

This passage contains a very proper hint on the necessity of distinguishing the red appearance which the coat of an artery acquires by long exposure, and that which occurs on its first examination. The former is imputed to the well-known effect of the air on blood, and the latter to the peculiar properties of the inflammation, namely, the presence of a greater quantity of arterial blood, and the increased number of small vessels carrying red blood. But the tyro will ask, why is this length of time necessary to produce such a change in the appearance of the blood? "I have seen it, (says Mr. Hodgson) in subjects that have been inspected a few hours after death, and cannot therefore regard it as produced merely by exposure to air." Now, it is well known, that a very short exposure to air is sufficient to give this red appearance to the blood. Why, then, should we not see in a recent subject, as well as "in arteries which have been long exposed in the dissecting room." No doubt, the author was so well aware of the cause as scarcely to think it necessary to explain it; but we, who meet with readers of every description, feel it our duty to give some opinion on the cause of this difference. In a recent subject, the blood is contained in vessels which are not pervious to the air, because they retain their life. That the air cannot produce this effect through a living substance, however thin, is evident, because we find, when stagnating in the lips of asthmatic people, it never acquires its crimson appearance. But that it will absorb oxygen through a much thicker membrane, and through a large quantity of serum, is evident by Dr. Priestly's experiment through a common bladder, and by the appearance constantly discovered, when blood taken from the arm is left to coagulate in a basin, and the red parts are covered by serum.

We

We submit it to Mr. Hodgson and our readers, whether we should not impute it to the permeability of dead animal matter to the air, whilst living matter resists it. Hence the change often perceived in the countenance of a dead subject. But the individual parts retain life after respiration has ceased, which is proved by the continuance of irritability; and, as long as this life continues, so long will the blood in the subcutaneous vessels retain its purple appearance. At length the parts, ceasing to live, are permeable to air, and the chemical properties of the air shew themselves in the altered colour of the blood.

"Arteries (continues Mr. Hodgson) are also subject to chronic inflammation, which lays the foundation of various morbid alterations in their coats. Chronic inflammation is generally to be observed in thickened and calcareous arteries, particularly in aneurismal subjects. In some instances it may probably be regarded as the effect of those diseases, although it is more probable that such depositions or alterations of structure are caused by increased vascular action. The internal coat of the vessel is soft, thickened, and of a deep red colour, which is not uniform, but irregularly disposed in the vicinity of ulcerations, thickenings, or calcareous depositions. This appearance in arteries has long been known, and accurately described by various authors, and the ancient physicians ascribed it to the effects of the morbid, acrimonious, syphilitic, and scorbutic humours that pervaded the system. Some modern writers also, and among others Scarpa, Corvisart, and Richerand, are inclined to impute it to similar causes, and particularly to the action of the syphilitic virus, or of the mercury that is used for the cure of that disease. From my own observation, I may remark, that I have, for the most part, found aneurism, and those organic alterations which generally attend the formation of aneurism, in subjects that have suffered much from venereal diseases, and who have taken large quantities of mercury. It appears, indeed, by no means improbable, that the internal coat of arteries may be one of those parts over which some specific diseases may exert their peculiar influence. Our knowledge on this subject is at least not sufficiently extensive to warrant the total rejection of opinions which derive some authority from observation, although our information is too hypothetical to be regarded as placing the subject in the condition of an established fact. It is, therefore, mentioned at present only as an object worthy of attention."

Whatever objections we may have to the above paragraph, the modesty of the conclusion is an example we shall keep in view and which we trust our remarks will not contradict. We do not recollect any ancient author who suggests a venereal cause in these diseases excepting Lancisi. But we know that, at one time, whatever could not be otherwise explained was referred to this as one common cause, and, in our our present eagerness to make amends for past errors, perhaps we impute as much to mercury. This would be well, if it led to a more exact inquiry into the effects of the disease and the remedy; but, is the practice of any of us yet freed from empiricism in the use of that mineral? where an artery has suffered so much violence as to be thickened with calcareous depositions in the manner described in the above extract, we should suspect that this mischief had been effected by an acute inflammation, the property of which in arteries is, we know, to produce the secretion of calculous matter. If the patient survives the inflammatory attack, the artery is very likely to assume this form, and the disease may now be called chronic, but we suspect its origin was acute. Ossifications, without any breach in the substance, we should rather consider the effect of chronic inflammation. It may not be amiss, in this place, to offer a conjecture on the cause of that disposition to secrete phosphate of lime in diseased arteries. Is it that their elastic coat partakes of the property of cartilage, and under inflammation takes on the ossific process, as we find in inflammation of bones; and does the covering membrane partake of that property like the periosteum? But may we not, also, account for the disposition in old people to ossifications in the larger arteries, without any apparent inflammation, by the well known property of cartilage to ossify; or has there been, in these subjects, an inflammation so chronic as to induce this gradual change without pain, and to which the parts accommodate themselves almost without inconvenience. The ossification of valves, which, being inelastic, are not likely to be cartilaginous, may be an objection to this. But are we sure that the hardness of valves is from a deposition of calculous matter?

The next section is *on Ulceration of the Coats of Arteries.*—An effect which, if not induced by external violence, we should always impute to previous inflammation, by which the texture of the artery was so much injured as to render suppuration the necessary consequence.

"Ulceration (says Mr. Hodgson) rarely takes place in an artery, the coats of which have not undergone some previous morbid alteration. It is not unfrequently met with around the circumference of calcareous, and in the centre of atheromatous depositions. The coats of an artery are occasionally so completely destroyed by such ulcerations, that the blood passes into the cellular membrane surrounding the vessel, which is expanded into a sac, and this is one of the modes in which aneurism is produced. Sometimes matter may be pressed from underneath the edges of such ulcerations, and I have seen a large ulcer situated immediately underneath the semilunar valves of the aorta, which contained a considerable quantity of pus. Pus, however, is rarely seen about these ulcers, because it is no sooner secreted than it is washed away by the stream of blood passing through the vessel. It is not improbable that the internal coat of arteries, during inflammation, may secrete pus without the existence of ulceration, as sometimes it happens in serous membranes. The current, however, through the vessel would prevent such a state being distinguishable."

Some very good remarks follow concerning the destruction of the whole substance of an artery in the ulcerated parts, producing apoplexy, if in brain, or hæmorrhage, according to the situation of the vessel.

We think it right to transcribe the section on Sphacelation of the Coats of Arteries, as a further means of illustrating the difference between the adhesive inflammation and the mere coagulation of blood.

"Inflammation, when confined to the internal coat of arteries, very rarely terminates in sphacelation, at least I have not been able to meet with the circumstance upon record, nor have my own observations afforded any instances of it. Arteries, however, are frequently involved in the sloughing of surrounding parts, in which case the blood generally coagulates in the vessel to a considerable extent above the line of sphacelation. This circumstance prevents the accession of hæmorrhage upon the separation of the slough: and, the coagulum being subsequently absorbed, the vessel contracts, and is ultimately obliterated. The cause of the formation of this coagulum is by no means evident, although it is probable that the condition of the mortified vessel interrupts the passage of the blood through it, and a coagulum is consequently formed, extending to the next important collateral branch. Amputation is sometimes performed a little above the line of separation in which it is unnecessary to tie the arteries, and parts destroyed by sphacelation generally separate without the occurrence of hæmorrhage, the cavities of the vessels being plugged with coagulum."

This is a process particularly noticed by Mr. Hunter, and produced among his proofs of the vitality of the blood, and that its coagulation arises from *the impression it receives.* In the present instance, he imputes the coagulation of the blood to the same laws by which the artery afterwards contracts, and the coagulum is absorbed, namely, to those powers of preservation "which, (to use the language of that distinguished pathologist,) evinces life in every part of an animal body, and renders every part of a living body susceptible of impressions which excite actions." In this manner alone he attempts to account for the coagulation of blood under mortification. "We know (says he) that blood will coagulate in the vessels themselves, and, under certain circumstances, sooner perhaps than any where else, when there is a disposition for mortification; in this case, the blood coagulates even in the larger vessels.

"I have seen (continues Mr. Hunter,) a mortification come on in the foot and leg, and, when it had advanced to a certain degree, the person died. On examining the parts above the mortified part, I found the crural and the iliac arteries filled with strong coagulated blood. We may thence infer, that the tendency to mortification in the vessel produced this disposition in the blood. If the coagulation should be supposed to have arisen from the blood being stopped in the larger vessels at the mortified parts, let us reflect that this cannot account for it. The same thing ought to happen in an amputation, and in any case where the large vessels are tied up."—By this, it is evident that the interruption to the passage of the blood is not necessary to produce coagulation in the vessels; it is well known that it rarely happens from that cause.

The fourth section is *of the various Morbid Appearances in the Coats of the Arteries.* Much of this has been already anticipated, particularly what relates to ulceration and the deposition of calcareous matter. Some appearances are, however, more minutely described.

"The internal coat (says Mr. Hodgson) of arteries is sometimes thickened and converted into a substance resembling cartilage, or the thickened peritoneum of an old herniary sac. If the coats of the vessel be separated, the disease will be found to occupy only the internal coat, which, having lost its elasticity, sometimes cracks, and forms scales that hang into the cavity of the vessel. This alteration of structure generally takes place to a considerable extent, and is frequently accompanied with a deposition of calcareous matter. The surrounding parts of the membrane generally exhibit the appearances of chronic inflammation, but I have never seen red vessels on that portion of the internal coat which had been converted into this cartilaginous structure. It is by no means so frequently met with as many other diseases of the coats of arteries."

These appearances are also noticed in the valves of the heart and aorta; and, to the difficulty in such cases of preventing the return of the blood, the increased size of the heart is very reasonably ascribed. Many other changes are marked with much accuracy, in which the structure of the artery is injured, and in most of which the author has discovered a deposition of calcareous matter. He conceives, that in some cases the internal coat remained entire, because he found a thin pellicle, between which and the muscular coat, the calcareous matter is deposited. We have seen this also, but have always suspected this pellicle to be coagulated lymph, lymph, deposited in the form of a membrane after the proper membrane has been destroyed. In those cases in which such concretions are attached by peduncles to the side of the heart, and hang like polypi to its sides, or where the pellicle hangs into the cavity of the vessels, and the blood is in contact with the calcareous matter, we conceive the effusion of coagulated lymph has been more irregular, or has been interrupted in its progress towards forming a membrane. Sometimes, it is remarked, the artery is obliterated, at others dilated and aneurisms formed. All these cases, we suspect, originated in inflamed arteries, and the consequent effects were in proportion to the degree of injury sustained by one or more of the arterial coats. On this occasion, however, we are obliged to notice one appearance which is described as similar to certain elevated ulcerations, or soft werts often found on the genitals, and commonly termed venereal. We suspect, that, in both cases, they arise from a similar, and in neither from a venereal cause; from their situation, the cicatrizing process is so often interrupted, that at last the part remains elevated by granulations, which assume a fungous appearance of different forms and degrees of firmness. Corvisart considers them as syphilitic, but Mr. Hodgson very justly doubts the accuracy of that anatomist, and produces a case in which syphilis could not have been the cause. Many judicious remarks occur on the cause of aneurism, to which we shall attend more particularly when that disease comes in order before us, and conclude our remarks on this chapter with the author's short observations on Angina Pectoris, or, as Dr. Parry more properly calls it, Syncope Anginosa.

"When the deposition (says Mr. Hodgson) of calcareous matter has proceeded to a considerable extent, irregular eminences project into the cavity of the vessel, whereby its calibre is diminished, and in some instances completely obliterated. The impediment which is from this cause opposed to the circulation, will explain the wasting of structure and deficiency of nervous power which occur in organs, the vessels of which have undergone this change. It is now universally known, as I have already observed, that a due supply of blood is essential not only to the nutrition, but also to the evolution of nervous energy, and consequently to the right performance of the functions of every organ. It is upon this principle that it has been proposed to intercept the growth of tumours, by diminishing the supply of blood by the ligature of their principal arteries. This practice was attended with some success in a case of bronchocele, in which Sir William Blizard diminished the supply of blood by the ligature of the superior thyroid arteries. It is also upon this principle that Mr. Hunter, and subsequently Drs. Jenner and Parry, ascribed the symptoms of angina pectoris to the deposition of calcareous matter in the coronary coronary arteries of the heart, whereby the stream of blood destined for the support of that organ is so much diminished, that its substance degenerates and wastes, its muscular power is lessened, and ultimately is insufficient for the purposes of the circulation. This condition of the coronary arteries does not, however, exist in every case which is attended with that train of symptoms to which have been applied the terms angina pectoris and syncope anginosa. Violent pain in the situation of the heart, extending down the arms, and terminating in a sensation of numbness, palpitation and irregularity in its action, with frequent syncope and difficult respiration, accompany almost all the organic diseases of that organ; at least, I have witnessed them in dilatations and aneurisms of the aorta, contractions of the valves, adhesions of the pericardium, and in active and passive aneurisms of the heart. Angina pectoris and syncope anginosa must, therefore, be regarded as terms designating rather a train of symptoms accompanying almost all organic diseases of the heart, than any particular morbid condition of that organ.

"That a degeneration and wasting of the muscular structure of the heart attends an extensive deposition of calcareous matter in the coronary arteries, has been noticed by Jenner,* Parry,† and Baillie.‡ The following cases exhibit the extent to which the muscular structure of the heart may be reduced from this cause, and prove how inadequate such a viscus must be to the due performance of its functions."

We have nothing to object to in the above extract, but that we are not directed to the passage in which Mr. Hunter ascribes the symptoms of angina pectoris to the deposition of calcareous matter in the coronary arteries, and the subsequent lessening of the muscular power in the heart. Mr. Hunter, in the only place that immediately occurs to us, in which he speaks of the symptoms of this disease, ascribes them, like Mr. Hodgson, "to a vast variety of causes." How far we are to attribute the diminished size of the heart to its want of nourishment from the state of the coronary arteries, is a question in which we shall not at present engage.

The succeeding section is on the *preternatural dilatation of the arteries*§. Here we have a description of dilated arteries, which are unconnected with aneurism. The most frequent seat, Mr. Hodgson observes, is in the ascending arch of the aorta. The coats of the artery, forming the boundary of the sac, are remarkably thickened and covered with atheromatous and calcareous depositions. He continues—

* Parry on Angina Pectoris. † Ibid.
‡ Baillie's Morbid Anatomy, p. 45.
§ Treatise on Blood, &c. p. 55.

"But

"But there is some degree of uniformity in this diseased state of the vessel, for the same morbid alterations are found in various parts of the sac; and this circumstance appears to me to prove that the coats of the artery compose the disease, for it is the internal coat in which calcareous matter is deposited; and, if we find that substance in all parts of the sac, it appears fair to infer, that the internal coat enters into its composition, inasmuch as that "identity of disease indicates identity of structure. This membrane, indeed, is much thickened, and resembles the peritoneum in an old herniary sac. Smaller sacs or pouches often grow from the sides of the great cyst, and are lined with a calcareous crust. At other times, the dilated coats appear to have given way at some point, and an aneurism is thus, as it were, ingrafted upon the dilated artery. This circumstance has, no doubt, tended very much to confound the disease with aneurism; but in such cases the part at which the neck of the sac commences is very evident, and its lining does not present those morbid appearances peculiar to the coats of arteries. There is also another circumstance in which this disease differs from most aneurisms, namely, that such dilatations very rarely or never contain any lamellated coagulum. Mr. Allan Burns has mentioned an instance in which some layers of coagulum were deposited; but the dilatation had, in that case, proceeded to such an extent, as to cause fissures in the coats of the sac in which the fibrine of the blood had been deposited. The dilatation often terminates abruptly at the arch of the aorta, but at other times it gradually diminishes. Sometimes the dilatation is partial, and occupies only one side of the vessel, which is expanded into a pouch, having much the appearance of an aneurism; but such partial dilatations very rarely contain lamellated coagulum. The essential differences between the two diseases will be considered when treating of the formation of aneurism."

This is contained in the succeeding chapter, which makes the first of the second division of the work.

"When the coats of an artery have given way from any of the causes detailed in the preceding part of this treatise, such as ulceration, dilatation, or rupture, and the blood passes into a cyst formed by the condensed surrounding parts, so as to be out of the course of the circulation, the disease is termed aneurism. Aneurism is also said to exist when an artery being wounded, and the integuments above that wound being united, the blood is effused into the parts immediately surrounding the vessel, and forms by its pressure a sac which has no external opening. There are other diseases of the vascular system to which the term aneurism has been applied, but at present I shall consider only that variety which is produced by the disorganization of the coats of an artery from an internal cause.

"SECTION I. *On the Formation of Aneurism in general.*—Notwithstanding the attention which has been given to this branch branch of pathology, there exists much uncertainty as to the nature of the parts which form an aneurismal sac. The question is simply this: Does aneurism ever consist in a general or partial dilatation of all the coats of an artery, or is it constantly produced by the destruction of all or most of these coats, and the formation of a sac by the influx of the blood into the sheath of the vessel and surrounding parts?

"The controversies which have existed upon this subject appear to have arisen from a reluctance to admit the possibility of more than one cause in the production of the same effect, and from an adherence to opinions deduced from very imperfect observations. As the ancients did not examine morbid appearances by dissection, their opinions on all subjects which, like the present, can be decided only by anatomy, must be merely speculative, and of no value. It therefore can be of little consequence whether Ætius and Paulus did or did not describe aneurism as formed by a dilatation of the coats of an artery, or whether they agreed with most of the other Greeks and with the Arabians in defining aneurism to consist in the rupture of an artery and the effusion of blood into the surrounding parts. Vesalius first applied anatomy to the investigation of disease, and he described an aneurism arising from the rupture of a dilated aorta. Subsequent examinations by Sennertus, Elseuerus, Silvaticus, Severinus, Hildanus, Barbette, and others, produced the doctrine that aneurism is universally caused by rupture of the proper coats of an artery. In the mean time, Fernelius advanced the doctrine that aneurism is produced by a dilatation of all the coats of an artery, similar to that dilatation which takes place in veins affected with varix. This opinion was supported also by Forrestus, Diemerbroek, and other writers. Further observations gave rise to a third opinion; and the cases recorded by Lancisi, Friend, Guatrani, and Morgagni, seemed to prove that aneurism may be occasioned either by the rupture or by the dilatation of the coats of an artery, or by a combination of both circumstances, the dilatation having preceded the rupture. Systematic writers adopted this doctrine with regard to the formation of aneurism, and the different varieties of the disease were distinguished by the terms—the true, the spurious, or the mixed aneurism. In the true aneurism, a sac was described as formed by a dilatation of the coats of the artery; in the spurious, the coats of the vessel were said to be destroyed, and the surrounding parts to form the sac; and the term *mixed* implied that variety in which the coats of the vessel were dilated to a certain extent, and subsequently by their destruction the true aneurism was converted into the spurious. Innumerable cases were recorded in which the sac appeared to be formed either by dilatation or rupture of the coats of the vessel, and upon these opinions and facts was grounded the doctrine generally received with regard to the formation of aneurism, until the publication of Scarpa's elaborate treatise. This indefatigable anatomist has revived the doctrine of Sennertus, and contends that aneurism is never produced by a dilatation, "but by a corrosion and rupture of the proper coats of the artery, and consequently consequently by the effusion of arterial blood under the cellular sheath, or any other membrane which covers externally the injured artery."

"Scarpa acknowledges the existence of that state of præternatural dilatation of an artery which I have described, and mentions the frequency of its occurrence in the ascending aorta. He details some of the circumstances in which it differs from aneurism, although he admits that the two diseases frequently exist in the same vessel; and observes, that they have generally been confounded under the same denomination. The circumstances however in which they differ are so remarkable, that in a pathological point of view they require discrimination. I shall therefore offer a few remarks upon the subject, before I detail the result of my own observations with regard to the formation of aneurism.

"The disease to which I allude consists in a præternatural dilatation of the whole circle of an artery, and not in a partial or lateral distension of its coats. The root of an aneurismal sac, however, never or very rarely occupies the whole circumference of the vessel, but commences on one side by a neck which is, in most instances, narrower than the body of the tumour. An artery is sometimes præternaturally dilated without any morbid alteration having taken place in the texture of its coats. On the other hand, the structure of most aneurismal sacs differ essentially from that of a dilated artery. The former in general possesses a smooth membranous surface lined with coagulum, and in an advanced stage of the disease it exhibits no traces of the coats of the vessel; whilst the latter possesses an uniformity of structure, and is evidently composed of the coats of the artery, which are generally in a morbid condition. The passage of the blood is not so materially interrupted in a dilated artery as when it passes into the recess of an aneurismal sac, and consequently grumous clots and lamellated coagulum, which are almost constantly deposited in aneurisms, are never met with in dilated arteries. These observations show that there is an essential difference between the præternatural dilatation of the whole circle of an artery and an aneurismal sac, whether it originates in the destruction or partial dilatation of the coats of the vessel.

"My own observations will not allow me to coincide with Scarpa, in defining aneurism to be constantly produced by a destruction of the coats of an artery. On the contrary, the inspection of innumerable preparations of this disease contained in the principal museums of this metropolis, and the more minute examination by dissection of various specimens of diseased arteries, and of aneurisms in the different stages of their formation, have produced a conviction in my mind, that, although in most aneurismal sacs, especially in those which have arrived at a considerable size, the coats of the vessel have given way, yet in a great proportion of aneurisms the disease commenced in a partial dilatation of the coats of the artery. The evidence upon which this opinion is founded will be detailed in the following observations on

2 the the formation of aneurisms, 1st, by destruction; and, 2dly, by a partial dilatation of the coats of the vessel.

"A great proportion of the aneurismal sacs which I have had an opportunity of examining, were unquestionably formed in the manner which Scarpa has described, namely, by a destruction of the internal and middle coats of the artery, and the expansion of the external or cellular coat into a sac. The cellular coat at length gives way, and the sheath of the artery and the surrounding parts form the boundary of the tumour. When the internal and middle coats of an artery are divided, and air or water is injected into the vessel, the external coat bulges very considerably, and constitutes a small aneurismal sac. Nicholls exhibited this experiment to the Royal Society by injecting water into the pulmonary artery with such a degree of force, that he ruptured the internal and middle coats, and distended the external. I have frequently repeated the experiment by dividing the internal and middle coats of different arteries by the application of a ligature, which being removed, and the vessel forcibly inflated, the external coat has always exhibited a sufficient degree of dilatation to prove that it is more liable to yield and to be expanded into a sac, than to be ruptured by the impulse of the circulating blood. It appears, therefore, that, when the internal and middle coats are destroyed, the sac is in the first instance formed by an expansion of the external coat of the artery: as the distension advances this membrane gradually gives way; the sheath of the vessel then restrains the effusion, and, yielding in its turn, the surrounding parts, whatever may be their texture, form the walls of the extravasation. The inflammation that is excited in the coats of the artery by the primary disease, and subsequently in the surrounding parts by their distention, produces amongst them an effusion of lymph which glues them together, and prevents the effusion of blood into the cellular membrane. Thus is the sac formed, and the inflammation which exists within it produces an effusion of lymph with which its internal surface is lined like that of an abscess.

"The causes which give rise to the destruction of the internal and middle coats of an artery, are ulceration and rupture. Ulceration, as I have already observed, is rarely met with in the coats of an artery which have not undergone some previous morbid alteration. It, however, frequently takes place in arteries the coats of which contain atheromatous or calcareous depositions. The internal coat first gives way: the destruction of the middle follows, and is accelerated by the infiltration of the blood amongst its fibres. The external coat becomes incapable of resisting the force of the circulation, and yields so as to form the sac of the aneurism. My friend Mr. Brodie favoured me with a diseased and thickened aorta, which exhibited these processes in a very early stage. The internal and middle coats were destroyed apparently by ulceration, and the external was expanded into a small pouch not larger than a pea.

"The formation of an aneurismal tumour, in the generality of cases, cases, is preceded by rupture of the internal and middle coats of the artery; the destruction of these coats by ulceration is a less frequent cause of aneurism. When the internal coat has undergone those morbid changes which I have described under the denominations of the steatomatous and cartilaginous thickenings, or when calcareous matter is deposited in its substance, it frequently cracks and hangs in scales into the cavity of the vessel. The disease is sometimes so extensive, that the middle coat becomes involved in it; the fissure extends throughout its substance, and its fibres are readily separated by the impulse of the circulation. The blood thus comes in contact with the external coat, which is dilated into a sac, in the same manner as when the internal and middle coats are destroyed by ulceration. The aorta of a lady, whose case I have already detailed, illustrated this stage of the formation of aneurism. The internal coat was throughout converted into cartilage, or covered with calcareous depositions. At the arch of the aorta there was a transverse rent about an inch in length, which had penetrated also the middle coat. The blood had insinuated itself between the middle and external coats, the latter of which was elevated into a tumour about two inches in circumference, and presented the appearance of a circumscribed ecchymosis. I conceive, that, had the patient survived, this condition of the aorta would have given rise to the formation of an aneurism. A similar appearance was observed by Nicholls in the examination of the body of George the Second. "In the trunk of the aorta, says Nicholls, "we found a transverse fissure on its inner side about an inch and a half long, from which some blood had recently passed under its external coat, and formed an elevated ecchymosis." The laceration of the internal and middle coats of the artery generally takes place during some violent exertion. Having lost its natural elasticity by the disorganization of its structures, the artery is unable to resist the impulse of the circulation; and hence patients generally date the commencement of their aneurisms from the occurrence of some accident or violent exertion. I have never, however, met with the laceration of the coats of an artery which had not undergone some previous morbid alteration, nor do I think it probable that any exertion which did not lacerate the surrounding parts could be sufficient to rupture the coats of a healthy vessel. Richerand says, that by violently extending the leg upon the thigh the internal and middle coats of the popliteal artery are ruptured, and thus he accounts for the frequency of aneurisms in the ham. I have several times repeated this experiment, but have never lacerated the coats of the artery unless the degree of violence was sufficient at the same time to rupture the ligaments of the knee, an event which can rarely be supposed to take place in those accidents to which the origin of aneurisms is attributed. Again, the experiments of Dr. Jones*

* Jones on Hæmorrhage, p. 125.

prove that the sudden laceration of the internal and middle coats of a healthy artery is not followed by the formation of an aneurism. An effusion of lymph takes place, which renders the vessel more firm at that part. Mr. Hunter and Sir Everard Home* also peeled off the external coats of an artery in a living animal, to ascertain whether a deficiency of these coats would be attended with a dilatation of the internal, but they found that dilatation was not the consequence, and the effusion of lymph which took place upon the artery rendered it more firm and less capable of dilatation than in its natural state. The constant existence of disease in the coats of every artery which is the seat of aneurism, renders it probable, therefore, that the loss of elasticity predisposes to laceration from causes which would not be productive of that effect in a vessel which possessed its healthy properties.

"The destruction of the coats of the vessel generally takes place in a transverse direction. Sometimes, more especially in the arteries of the third order, the whole circle of the vessel is separated, and in other instances the division is partial, and the artery appears as if it had been opened by an instrument. Sometimes the destruction of the coats takes place in a longitudinal direction; sometimes it has a circular appearance, and its edges form a partition between the cavity of the sac and that of the artery.

"Cases in which aneurisms appear to have been produced by destruction of the coats of the vessel, abound in surgical and pathological writings. It is sufficient, however, in confirmation of the facts which I have stated, to refer to those recorded by Scarpa, Morgagni, Lancisi, Guattani, Desault, Warner, and Home, and to the plates of Scarpa and Guattani.

"But is every aneurism produced by a destruction of the internal and middle coats of the vessel, and does not a partial dilatation of these coats occasionally precede and give rise to their destruction? I believe that this is frequently the case. We have seen that the disorganization of the coats of an artery by destroying their natural elasticity will give rise to a permanent dilatation of the whole circumference of the vessel; and there is every reason to expect that a loss of its elasticity in a portion only of the diameter of the vessel, will give rise to a partial dilatation of its coats. Indeed the proofs of a partial dilatation of the coats of an artery, particularly of the aorta, are incontestably established by the possibility of tracing the coats of the vessel throughout the whole extent of the expansion, and by the existence of those morbid appearances in the sac which are peculiar to the coats of arteries.

"In the year 1811, I dissected an aneurism of the aorta, which was removed from the body of a young woman by my friend Dr. Farre. The sac was as large as a small melon, and had proved fatal by bursting into the posterior mediastinum, and subsequently

* Transactions of a Society for the Improvement of Medical and Chirurgical Knowledge, vol. i. p. 144.

into into the cavity of the thorax. This aorta exhibited the formation of aneurism by partial dilatation in three distinct stages. The internal coat was throughout inflamed, and presented a fleshy and irregular appearance. At the arch of the aorta there was a dilatation not larger than the half of a small pea. About two inches lower in the same vessel was a second dilatation which would have contained a hazel nut, and immediately above the diaphragm was the large aneurism which had proved fatal. I removed that portion of the vessel which contained the smallest dilatation, and macerated it until its coats could be separated without violence. I found that the dilatation existed equally in the three coats of the vessel, and, when separated, each presented the appearance of a minute aneurism. The second dilatation exhibited the same circumstances in a more advanced stage. The coats of the vessel were more intimately adherent to each other than in a natural state; but it was evident that the sac consisted in a dilatation of the internal, the middle, and the external coats of the aorta. In the large aneurism, the disorganized internal and middle coats could be traced for some distance into the sac, when the parts contained in the posterior mediastinum and the vertebræ formed the remainder of the cyst. There can be little doubt that this sac commenced in a dilatation of the coats of the vessel similar to those appearances which existed in the superior portion of the artery, and the dissection appeared to illustrate the formation of aneurism, by partial dilatation of the coats of the artery in three distinct stages."

We have given this long extract that we might not interrupt the author unfairly. We shall be very short in our remarks. To us, we confess, that the existence of aneurism, without the previous destruction of some part of the internal coat of an artery, is still problematical. The distinction between a dilated artery and an aneurism, was, we believe, first made by Scarpa, and we find it extremely difficult to mark the difference without admitting of Scarpa's conclusion. "The constant existence of disease, (says Mr. Hodgson,) in the coats of every artery which is the seat of aneurism, renders it probable," &c. If we admit constantly disease, we cannot easily assert that this disease did not exist before the aneurism was formed; and, if the internal coat appears perfect, we cannot assert that it has not been renewed by coagulated lymph, especially when we find calcareous matter below. Whether, in the case described at the conclusion of our extract, the upper dilatations would have become aneurism, we cannot ascertain, as Mr. Hodgson has not mentioned whether they contained any strata of lymph which he conceives characteristic of aneurism. Perhaps it will be found that the distinction is less important than it may seem, as all parties must, we conceive, admit that the artery is diseased before it it has lost its elasticity. The construction of an artery to preserve its form by the elastic power conjoined with the muscular, is so beautifully described by Mr. Hunter, that we felt perpetually disappointed in finding so little notice taken of the labours of that wonderful physiologist. In referring to page 129 of his Treatise on the Blood, it will be seen how accurately the whole question of elasticity and dilatation had been examined by him. The fair inference of all his observations would lead us to conclude that, as long as the artery retains its original elastic powers, the force of the heart can never produce any partial dilatation; that, whenever it loses its elastic power, it must either dilate or give way. In the first case we shall have the dilated artery, in the second the aneurism.

From a careful examination of Dr. Jones's experiments, it is not very difficult to conceive, that injury, similar, excepting in degree, may produce aneurism, or dilatation, or obliteration of the cavity. For a further account of these experiments, we refer our readers to Vol. XVI. p. 89, of our Journal. By these he will find that the degree of division of the internal coat becomes the stimulus to the future action of the vasa vasorum of the surrounding parts, and of the blood itself. It will there also be found, "that the sudden laceration of the internal and middle coat of a healthy artery is followed for the most part by an effusion of lymph, which not only renders the artery firmer, but in many cases obliterates it altogether. Mr. Hunter and Sir Everard Home's experiments are sufficient to shew, that mere weakness from mutilation without disease in an artery, may be repaired before dilatation takes place, consequently, that the latter can only happen when the artery is so far disorganized that its elasticity is irrecoverably lost.

We have made these remarks not with any wish to detract from Mr. Hodgson's merit, of which we are highly sensible, but to shew, from the magnitude and complicated nature of the subjects, how necessary it is minutely to examine all their bearings, and whatever has been done before us.

The author next proceeds to the spontaneous cure and medical treatment of aneurism; a section which abounds with good sense and close observation. Of the spontaneous cure under different circumstances, the author gives many interesting, and we may add consolatory, cases. His proposals for medical treatment are not less judicious. The general conclusion drawn from this division of the work is—

"First. The deposition of coagulum in the cavity of the aneurismal sac and the artery leading into it, is the mode by which the spontaneous cure of aneurisms is, in most instances, effected.

 "Secondly.

"Secondly. The coagulum is subsequently absorbed, and the sac and the artery contract until the one becomes an impervious cylinder, and the other a small fleshy tumour.

"Thirdly. In some instances the cure is effected by the obliteration of the cavity of the sac without any obstruction taking place in the calibre of the artery from which the disease originates; in this manner a cure may take place in aneurisms of the aorta.

"Fourthly. The formation of coagulum being a general occurrence in aneurisms, it is an important object to prevent the increase of the sac, that the deposition of coagulum may proceed to such an extent as to obliterate its cavity.

"Lastly. It is the force of the circulation which causes the enlargement of the sac and its ultimate rupture; hence the diminution of the force of the circulation is the principal indication in promoting the spontaneous cure of aneurisms."

A section follows *on the Surgical Treatment of Aneurism, and on Collateral Circulation.* This is necessarily very minute, and we may add very judicious; we need not inform our surgical readers that it will not admit of being epitomized.

Having thus gone through the general process of disease in the arteries, and considered the various causes, progress, and diagnosis of aneurism, with the mode of treatment, Mr. Hodgson proceeds to offer a section on each kind of aneurism as distinguished by name and situation, on aneurisms arising from wounded arteries, and on the varicose aneurism, as well as aneurismal varix.

The fourth part is *on the Diseases of Veins;* on Inflammation; on various Morbid Appearances in their Coats; on their Obliteration and the consequent Collateral Circulation; on varicose Veins, Cirsocele, and Hæmorroids, which last the author conceives arise sometimes from the veins. If we concede this to him, we are not prepared to admit that any of the periodical bleedings from those parts are venous, because in all the cases we have seen, which are numerous, the appearance of the blood at its first discharge is truly arterial. This is, however, a mere matter of opinion, and of little importance.

An Appendix follows, replete with interesting matter, not easily introduced in the work itself. The first paper is an account of worms found in the arteries of some animals. These are principally in horses and asses. The latter have been examined by the author with much attention. His description is very minute, and, we doubt not, equally accurate. We agree with him, that the accounts of worms in the human arteries are not satisfactory; but we could refer to other testimonies of worms found in the heart of dogs dogs of the description mentioned by M. Peysson, and also to a living witness, who found them in several dogs during the time that an epidemic disease prevailed in that race. It is not certain that the worms were the cause of the disease, as they were not found in all that were examined.

A very interesting case follows, in which there was an obliteration of the brachial, radial, ulnar, femoral, popliteal, and tibial arteries.—A case of popliteal aneurism, cured by the obliteration of the femoral artery, under the use of Professor Assalini's forceps, of which a drawing will be found in page 2 of our last Number. This case we have given in our Collectanea.—A Case of carotid aneurism; a case of axillary aneurism, by Mr. Thomas Blizard, in which the subclavian artery was tied. This we have also reserved for our Collectanea, as it is, we believe, the first instance in these important operations in which the patient lived long enough to shew that the collateral branches were insufficient to carry on the circulation in time to preserve the most extreme parts. The operation and the relation of it does equal honour to the judgment and candour of that distinguished surgeon.

Such are the contents of this valuable performance: as we have not been backward in offering our opinion of every part particularly noticed, we have only to remark, that the whole does great honour to Mr. Hodgson's industry and genius. We have no doubt that the book will be considered a text book, and be referred to in all subsequent experiments, as well as operations on these delicate organs. In some parts, if we do not think with the author, our remarks should be rather considered as difference of opinion than real objections. We regret that the work must be so expensive, on account of the numerous well-executed engravings; and we cannot help adding, that we have found some of the descriptions quite sufficient, which the author has thought it necessary to illustrate by drawings.

---

*A practical Explanation of Cancer in the Female Breast, with the Method of Cure, and Cases of Illustration.* By JOHN RODMAN, M. D. one of the Surgeons and Medical Superintendants of the Dispensary, and House of Recovery of Paisley. 8vo. p.p. 240. 1815. Underwood.

THE object of the author seems to be an attempt to reduce the causes of Cancer to a few simple principles, to prove the locality of the disease, and to disprove the existence of what has been termed a cancerous diathesis. A careful perusal of the work, however, has not enabled us to perceive perceive the success of the attempt, or to admit many of the cases stated by the author to have been cases of cancer; yet several of the diseased breasts, which came under his care, had been condemned to the knife by preceding surgeons. The facility with which some of these were cured by simple applications, proved, indeed, the ignorance of surgeons who could do nothing without the knife, but it by no means established the cases as instances of cancer, or justified the author's scepticism, we would almost say, disbelief, of the existence of the disease as one differing in its nature, appearance, and symptoms, from any other disease.

He commences with some observations on the *Feeble Structure of the Female Constitution*, of which the following is a specimen.

"The pursuits of life present a vast mixture of inducements by which mankind is allured; and the female sex display great anxiety to occupy their several departments with activity. The strength of every individual, however, is not alike suited for the continuance of this activity, and many females pine under the exertion, with marks of disease and evidences of lingering constitutional frailty. Yet *nevertheless*, of such disadvantages, some survive the ordinary periods of existence under calm composure and steady deportment, while others fall victims to premature disease, the consequence of negligence, and the result of delicacy."

We extract no more of this part of the work, because the remarks, though sensible enough, are such as would probably occur to most practitioners, and do not apply to cancer more than to many other diseases; yet the author deems them very important, for he concludes—

"Probably the reader, by this time, after what he has himself observed corroborative of what have *(has)* been here stated, will be enabled to remove the veil and see the fallacy of the opinion that latent powers, hidden malignancy, or occult poison, pervade the system in the production of mammary cancer; and will be inclined to lay aside all the theories that are founded upon such speculations, particularly if his own observations confirm the subsequent statements, which, along with the cases, are intended to shew how local affections result as ordinary consequences."

The chapter on the "*disposition of mammary glands, and origination of tumours*," contains some judicious remarks; but they apply simply to inflammation of the parts, and not to cancer. The chapter on "*the locality of mammary disease, arising from affections of the mind*," is written to establish a point which no one will dispute, that females are apt to imagine themselves afflicted with cancer, especially when they have witnessed the disease in their friends, or have been reflecting or conversing much on the subject. In some individuals, the influence of the imagination has even been supposed to to produce a tumour in the breast, with pain, and some of the symptoms of cancer; but in general such affections yield to mild treatment. The author has adduced two cases in support of the opinions advanced in this chapter; we shall state them in his own words, though we do not regard them as cases of cancer; they, however, serve to illustrate the effects of fancy or imagination.

"An unmarried lady took suddenly ill, and, though her complaints were very irregular, she had sickness, head-ach, quickness of pulse, interrupted breathing, and symptoms of general agitation for some time, which seemed unaccountable; also several other ailments, occasionally, that varied much in the way of affecting her.

"She continued ill for a number of months, and her health was restored at last by sea-bathing, and agreeable society, when I obtained the following information.

"Previous to the illness, a female friend, while conversing with her, had entered into a long detail about some cancerous mamma, and had told her all she knew concerning its amputation, the appearances, the sufferings, and the medical remarks; the whole forming a history which was particularly fitted to produce feelings of horror in the female mind.

"The friend was not long absent after giving this narration, when the lady began to digest the leading objects of the story, and the occurrence of darting pains had been a part of the subject which was chiefly canvassed. She brooded over all the circumstances with serious interest, and, the more they occupied her attention, the distresses were the more magnified in her thoughts, till, in a few hours, she began to feel darting pains entering into her own left breast, with a considerable degree of uneasiness through all the gland.

"Though no former disease had been felt in this breast, it occurred to her, that cancer had, perhaps, been forming there without her knowledge, and that she might have been still ignorant of it, unless she had received the recent information of darting pains being particularly noticed by medical men as marks of the distemper. Her alarm went on, her stomach was soon affected, costiveness ensued, and the breast became swelled and troublesome.

"Thus, while disease was growing, and she experienced an increasing frequency of mammary pains, her resolution to conceal them, and never to speak of such an affection, became the stronger; and her reason was, that she would never submit to have her breast cut off, which she thought, if a medical man were told all her sufferings, would be his first advice, because no other thing could be done.

"In this way she struggled with a series of fears and hopes, keeping the primary cause of affliction to herself. Yet, although the disorder of the breast gradually abated, and ceased in about two months, the consequence was great debility of body, accompanied with such an irritability of mind, that she could not divest herself of afflicting thoughts which distempered her frame for a long time after.

"A lady consulted me in the year 1797 for an oval tumor in her left breast, as large as an ordinary plum, which arose from the accidental stroke of a man's elbow. She had been three months troubled with it before I was applied to, and the whole gland was exceedingly tender, with general swelling, and tense tumid lymphatics.

"Her habit of body was delicate, and she was subject to sickliness and indigestion, which occasioned other complaints, and generally augmented the uneasy feelings in the mamma. But, whenever a temporary fever was induced by cold, the mammary disorder became painfully worse, and soon appeared as the chief morbid affection. The treatment first adopted was leeching, when local inflammation was strongly indicated, and general blood-letting, when it became necessary for other affections of the system, that quickened the distemper within the breast.

"If renewed inflammatory appearances afterward in the breast were moderate, chamomile fomentations and cataplasms became particularly useful. They generally reduced the swelling, and caused an abatement of painful sensations, when resorted to at the commencement of such ailments. The application of warm vinegar, camphorated spirits, and sometimes laudanum, frequently proved serviceable. From time to time she likewise used medicines for the purpose of strengthening the digestive organs, and preserving a soluble state of the bowels. Little persuasion was required in order to force attention to these means. Uncommon weakness in the stomach, or a considerable increase of costiveness, soon revived the uneasiness in the mamma, and that uneasiness she carefully noticed. Other local means and plans of an invigorating nature were persued according to circumstances. But the method mentioned conveys a general view of the mode of prescription, along with pointed care for the preservation of proper warmth in the body, and in the diseased breast, which was not neglected. Within three years the disease was so completely subdued in this way, that little evidence of a mammary tumour could be traced. The body of the gland was only somewhat delicate, and more easily disordered by injurious casualties than the other breast.

"In 1809, after the mamma had been long healthy, a female whose welfare interested the lady greatly as an intimate friend, had a breast amputated for a distemper which was pronounced inveterate cancer. Immediately upon hearing of this event, the lady recollected that there was a similarity of the two disorders about ten years back, and having seen the female not long before the operation without any thing of the kind being spoken of, her alarm was instantaneous. A sudden pain assailed her formerly-diseased breast, and soon rendered her incapable of attention to assist another person in like circumstances. She busily contrasted the complaints and the result; and, because of the violent pain which was fast increasing, she had no doubt of speedily suffering under the same kind of operation, and endeavoured to bring her mind into a state of submission. As was to be expected, this attempt increased the evil, and she became inconsolable. Painful swelling of the breast came on to an alarming degree, and I remarked that the other breast gave her no uneasiness even under such mental agitation. The disturbance appeared only in the breast which continued to be the subject of her fear.

"During this state of mind, the whole glandular substance of the mamma became firm and bulky. Lancinating pains were frequent and severe, returning at times with such violence, that close conversation with a stranger in company could not prevent the rapid and involuntary application of her hand upon the affected breast. In this manner the disease advanced for several days, and all my endeavours to mitigate her distress proved unavailing, until I gained an ascendency over her perturbed imagination, and brought her to credit my protestations that there was positively no cancer in the breast. After obtaining her confidence in my assertions, composure of mind ensued, the swelling of the breast diminished, and the pains gradually abated. The remedies which she had used to little purpose when the mind was agitated, were now effectual, and removed the disorder. Topical warmth was strongly recommended, as being necessary for some time after, and in a few weeks all was well.

We agree with the author in his chapter on "*Indurations or Tumours of the Female Breast*," that there is sometimes great difficulty in distinguishing scirrhus from some other tumours to which the organ is liable. The chapter on "*the Influence of Cold*" conveys little instruction, except to those learned professors, who are able to construe the following paragraph.

"The principles on which to explain how cold disturbs the powers of digestion, are much the same *of* those *on which* to observe how cold distempers the female breast; because, if the disease of the gland be such as that which is mentioned in the first section of the second chapter, cold impairs digestion according to the principles just mentioned, and infects the breast at the same time."

In the succeeding chapter, the author recapitulates what he has advanced in the preceding chapters, and states several cases; of these we shall extract two or three in the order in which they stand, without any selection.

"*February* 23d, 1810.—A female about 20 years of age consulted me to-day for a tumour in her left breast. The pains in the breast are not very severe, yet they are frequently of a darting nature. The tumour is as large as an ordinary sized plum, and has been lodged in the gland for a long time. She seems naturally to possess a calmness of mind, and, although it is evident that she is alarmed, in some degree, for the disease, because she was told it was cancer, there is a considerable appearance of composure in her countenance.

"She

"She informed me of a soreness in the left arm-pit, and, having examined it, I found two bulky glands there, but could not find neither disease nor swelling in the right breast or arm-pit; at the same time, I observed a scar on her neck, the cicatrice of an old scrophulous ulcer.

"She belongs to the West Highlands, and has been accustomed to subsist on very meagre diet. Her employment is sedentary, (at which she is confined from twelve to sixteen of the twenty-four hours), and her complexion is very unhealthy.

"I advised her to the use of food, more nourishing than what she had been accustomed to take; and pointed out the benefit she would derive, from engaging in some occupation which might require moderate activity, and less confinement. She spoke of an opportunity she had of this kind, in the neighbourhood of a medicinal well, and I recommended that she should go there speedily, stating the advantages of proper clothing, and the necessity to preserve the diseased breast in a regular state of warmth.

"*April* 21st, 1812.—An intimate friend of hers called, by desire, to tell me that she pursued the advices respecting exercise, diet, and local warmth, and, in a few months, the mammary tumour vanished, as well as the tumours in the arm-pit. It appears that she is now strong and healthy, seeming altogether like a different person from what she was at the time of consultation.

REMARKS.

"This case affords an instance of tumour in the female breast, while a scrophulous affection might be said to exist in the system; yet she knew of no such disease in her family, and it was in very early life that she had the ulcer in her neck. At any rate no disease of this kind was spoken of by others who had examined her, and I might have remained ignorant of it also, had not a chance circumstance occurred by which I noticed the scar.

"*Nov.* 23d, 1809.—A servant girl, 18 years of age, desired my advice for a tumour in her left breast.

"She was struck accidentally upon the breast about a year ago, and it continued painful for a week or two; after which she discovered a tumour in the gland, the size of a hazel-nut, though, till lately, she could not complain of having experienced much trouble from it.

"It is three weeks since she was overheated and fatigued, when the breast became uneasy; and, while in this state, she heard of a woman whose breast was cut off for a complaint of the same nature. As soon as possible, after hearing of this, she went to the surgeon who had operated in the woman's case, and was told, that the tumour was not the worst kind of cancer, yet, as it was doubtful how long it might remain so, her safety would be secured by allowing the operation to be performed pretty early.

"She had no idea that a business of this kind was to take place so soon, for the pains in the gland were very moderate. Nevertheless they increased after this advice, and, now, although six days since the advice was given her, she is evidently disturbed in her mind.

 "Upon

"Upon examining the breast which is swelled, but not greatly, I find the tumour in the substance of the gland, immediately above the nipple. When I press the gland laterally, the tumour feels bulky by the interposition of glandular substance, but by pressing directly down upon it, the size is trifling, only the substance around it is firmer than usual. There is no other affection that deserves to be noticed.

"From the good state of her health, I had every reason to assure her that she had no danger to fear, provided she covered the breast with one or two folds of flannel, and took care to bathe it frequently with warm vinegar. These advices she readily engaged to follow, and seemed determined carefully to avoid every chance of injury either by pressure or otherwise.

"*Dec.* 18th.—Her breast is quite free from pains, and the glandular swelling is gone—the firmness of the glandular substance is greatly dispersed, and the site of what was thought to be a tumour is perceived with difficulty. Fifteen months after this time, she had no complaint of the kind.

"Mrs.——— has been twelve months in a state of nervous weakness, with fluor albus, general debility, frequent uneasiness about the region of the stomach, and little appetite for food. Her circumstances in life have been adverse, and her spirits are usually depressed. It is six months since she discovered a painful tumour in her left breast, for the treatment of which a surgeon has attended her occasionally, during a great part of that time. But, on finding that no remedy proved effectual, and that the mammary disease was still increasing, he seemed alarmed at the nature of her complaints, and proposed to save her by the amputation of her breast. She applied to me on July 5th, 1813.

"The tumour is lodged in the upper part of the gland—it is felt like a knobbed body, but can be separated into distinct portions, by careful examination. All the substance of the breast is firmer than natural, and there are two bulky glands in the axilla, though I do not think them very hard. The breast is swelled, painful, and tender—the pains are often excessively severe and darting. Her mental feelings appear as if they approached more to a melancholy, than to a hurry of alarm. The other breast is firmer in some parts of its texture, than that which is common in a state of health, and it might be thought to contain a number of tumours. The menses are expected in a few days—she is very costive, and feels the effects of cold in the left side of her body, in a very particular manner.

"R. Ol. ricini semunciam. Sig.—The whole to be taken in the afternoon.

R. Sulph. magnes. unc. duas.
Pulv. rhei drach. un.
Carb. magnes. drach. duas.
Aq. pur. libras duas. M. Sig.—A wine glassful to be taken on the second nights.

"The

"The breast to be fomented daily with a decoction of chamomile flowers, and covered attentively with cotton wool.

"*August* 9th.—She has had a nervous fever since last report, and went a few days to the country, after recovering from it. The disease of her breast is mild, and the pains are greatly abated. The same treatment of the breast to be continued.

R. Carbon. ferri drach. duas.

Sacch. alb. drach. un. M. et in doses duodec. divide. Sig.—One to be taken at night and another in the forenoon. The bowels to be kept open as before.

"*Sept.* 4th.—The swelling and general painfulness of the breast increased—numbers of bulky glands to be felt through its substance—sickness and languor of the whole body. She fatigued herself too much for three days last week, and, since that, her feelings have been of a feverish nature, and her breast has been more uneasy.

"Take half a drachm of the Pulv. Cinchon. twice a-day, and two of the Pil. Rhei Comp. each night.

"R. Tinct. Sapon. cum. Opio. Sig.—The breast is to be rubbed with this, and covered as usual.

—— 25th.—Greatly better. The substance of the breast loose and soft, without pain—the hardened glands dispersed—the original tumour diminished in size.

*Oct.* 18th.—Is still better, and has been taking sometimes the bark, and sometimes the steel-powders. No swelled gland to be felt in the arm-pit, and, as for the mamma, there is no disease in it which deserves the name of a tumour.

"She is still to treat the breast carefully, whenever it becomes more painful, at any time, or bulkier than ordinary.

Several other cases might be extracted to prove the extreme ignorance and culpable precipitancy of several surgeons, (we presume in the author's neighbourhood), in deciding upon the necessity of extirpation, before they had tried proper remedies for restoring the breast to a healthy state. In this respect Dr. Rodman has great merit; but it appears to us, that he has seen very little of real cancer, and we think it may be inferred, from the general tenor of his observations, that he does not believe in the existence of the disease so denominated; neither should we, had we witnessed only such cases as those which he has detailed, with a disposition to regard pain, swelling, and induration of the breast, as a simple disease produced by various causes. We have no doubt the author has performed many cures, and been of much service in the district in which he resides; but we appeal to his candour, whether, with his particular notions of the disease, he has acted fairly in bringing forward such affections under the term of cancer? especially, too, when he does not scruple to avow that "the word cancer is a name, likewise, without any kind of true meaning; for, whatever it may be thought to imply, it leads into nothing but error."

error." His description of the disease also is extremely defective: having arrived at the chapter in which he treats of the cure, he finds that "it is necessary now to describe the disease for which a cure is to be proposed. Let it be spoken of as a hardening tumor and induration, or a bulky gland in the female breast, accompanied with vascular irritability, and painful turgescence of the part."—If he can succeed in persuading women thus affected, that they labour under scirrhus or cancer, he may indeed have patients enough, and readily convince them that cancer is a bugbear.

In useful practical works, we seldom animadvert on the style in which they are written; but this volume, small as it is, we are sorry to observe, abounds in instances of bad grammar, obscure phraseology, and Scotticisms which would not be indured at Edinburgh, or at any seat of learning in Scotland.

---

*Reflections on Fever: intended to point out the Principles upon which a systematic and useful Method of Treatment might be established.* By ROBERT CALVERT, M.D. of the College of Physicians, London; Physician to the Forces, &c. &c. &c.—8vo. pp. 84. Callow; London, 1815.

IF the author had not informed us that this work was written in the intervals which an itinerant life affords, we should have supposed it to have been composed as a school-exercise, or thesis, at the university where he graduated. The hypothetical so much predominates over the practical character of the treatise, that we cannot conceive it to have been the fruit of camp-experience, or the result of arduous duty in military hospitals. It is, however, an ingenious production, and calculated to puzzle other heads than those of reviewers, some of whom shrewdly pass over what they do not comprehend.

The nature of fever has long perplexed us, but Dr. Calvert at last has loosed the gordian knot, and that with so much facility, that he is necessarily surprised at the ignorance of preceding writers on the subject, and astonished that "the disease has never been accurately defined." Without quoting at least half of the work, which only consists of eighty-four small pages, we could not do the author justice in explaining his notions of fever. We shall, however, make a few interesting extracts by way of preparing the reader for the grand discovery.

"The pulse is one of those indices in fever that never goes unnoticed, although very few, if any, understand the laws by which it is governed. What inference can any one draw from feeling the pulse, who is both ignorant of its laws, and of the nature of the the disorder of which it is supposed to be the index? or what knowledge can we obtain from being told, that the pulse is *slow*, *quick*, *soft*, *hard*, *regular*, *irregular*, *running*, *intermitting*, *feeble*, *full*, *sinking*, *getting up*, *rising*, *falling*, *frequent*, *jerking*, *small*, *wiry*, *strong*, *weak*, *languid*, *vibrating*, &c.? During the progress of a febrile paroxysm, Dr. Cullen says, in the cold stage it (the pulse) is *sometimes slower*, *but always weaker*, *than before*: it then becomes *smaller*, *very frequent*, and *often irregular*: afterwards it becomes *more regular*, *hard*, and *full*: then it becomes *softer* and *less frequent*; and, finally, *it returns to its usual state*. (First Lines, ch. i. 12.) Another author, however, says, 'Pulsus (febriculorum) sanorum pulsui non admodum absimilis.' (Sydenham Op. 505. 519.) Dr. Irvine found the pulse, skin, and bowels natural; and in another instance of fever he found it beating only forty-two in a minute. (Some Observations on the Diseases of Sicily, &c. p. 13. 64.) In some bad cases of plague too, Russell found the pulse not much different from the natural state. (Russell on the Plague, p. 86. 87; also in Cases, No. xii. xxvi.) These examples, I think, are sufficient to prove that no useful inference whatever can, in general, be drawn from this adventitious and unsteady symptom."

It surely is unnecessary for us to offer any comments on this strange deduction from a very few particular instances, opposed to a general rule.

We pass by the pages on *causation* and *succession of events*, though we doubt not the soundness of the author's philosophy, and that the doctrine is essential to his system. We are next favoured with an account of certain laws that belong to the animal economy, from which it appears that there is an equilibrium between the ingress and egress of the circulating fluids; and this the author terms the *balance of circulation*.

"Sanctorius, and his commentator, Gorter, considered the balance between the ingesta and egesta of the whole of the animal juices as the standard of health (Gorter, de Perspirat. Insensib. cap. i. iii.); but, as the excrements do not enter into the circulating mass, they being merely to the animal what soil is to the plant, I exclude the contents of the intestines wholly from the system I am about to consider."

Excrements, blended with mould or other matters, may form good soil for plants, but we never before heard of their "being merely to the animal what soil is to the plant," i. e. nutritious.

The following extracts immediately precede the explanation of fever, and are necessary to understand it properly.

"It is evident that the balance of circulation may be lost in two different ways, viz. on the side of depletion, when the egress exceeds exceeds the ingress; or it may be lost on the side of fulness or plethora, when the ingress of fluids exceeds the egress.

"Depletion, again, may be occasioned by preternaturally diminished ingress of chyle; by preternaturally increased egress of excretions; by both conjoined.

"Fulness or plethora, on the other hand, may arise from preternaturally increased ingress of chyle; from preternaturally diminished egress of excretions; from both conjoined. I shall take examples of each of these peculiar states of the vascular system, in order to ascertain which of them, if any, belong to a febrile state of the body.

"Depletion, arising from diminished ingress, takes place in obstructions of the thoracic duct, impeding the flow of chyle. It happens in those who abstain from food and drink. Depletion occurs from excessive egress in diabetic patients—in excessive lactation—during the menstrual period—and from all natural and artificial hæmorrhages. Depletion, again, occurs from diminished ingress and increased egress conjoined; in jockeys, who rigidly abstain from drinking, while they create a copious flow of perspiration by means of exercise and external heat. In none of these examples, however, do we find that particular train of symptoms we call fever.

"As the process of absorption from the intestines is concealed from our view, it is difficult to prove that this is, at any time, carried to excess. It is extremely probable, however, that this happens during a constipated state of the bowels, while much is drank, and while there is no corresponding increase of excretions. This state of the body, every one knows, is a common attendant on fever. 'Si vero cibi maneant in ventriculo ampliore tempore quam opportunum est, et alii ad ipsos incidant corpus utique repleatur: et, dum *premuntur a plenitudine venæ, calor* ac *dolor* corpori accesserint, æstate quidem citius, hyeme vero posterius.' (Hipp. de Morbis, lib. vi. 17.)

"The next example that presents itself is, when fulness of the vascular system arises from diminished excretions; and I shall go no farther than Dr. Cullen's First Lines (part 40) to prove that this very frequently happens in fever.—'It is to be particularly observed (says he), that, during the cold stage of fever, there seems to be a spasm induced every where on the extremities of the arteries, and more especially of those upon the surface of the body. This appears from the suppression of all excretions, &c.

"Lastly, we shall have plethora arising from excessive ingress and diminished egress combined in all those fevers attended with costiveness, excessive thirst, and diminished excretions. From the whole of these examples, I think, we may fairly infer, that fever consists in the balance of circulation being lost on the side of fulness or plethora.

"The balance, however, may be lost in this way to a certain extent, without occasioning inconvenience, owing to the elasticity of of the vessels. This, indeed, must happen, more or less, in every case of fever, previous to the declaration of the symptoms. Or, should events occur in time to interrupt the accumulation, and restore the balance, the perceptible symptoms of fever may never occur at all. This state of the vascular system, however, in my opinion, ought to be considered as latent fever, the repeated attacks of which might explain the consequences we frequently observe in those people who have lived long in unhealthy situations, although they themselves have never been conscious of an attack. The repeated influx of fluids to the internal parts distends the vessels of those parts, and gives rise to those subsequent enlargements of the liver, and spleen in particular, that are so common in those situations. The symptoms of fever then are mere indices of the extent to which the balance of circulation has been lost."

Having thus easily determined the nature of fever, the author proceeds to explain more particularly "the effects of diurnal changes of temperature upon the vascular system, as tending to produce a febrile state of the body." As this explanation is altogether beyond our comprehension, we shall state it in the author's own language, professing that we know as little about a *diurnal flux and reflux in the vascular system*, and *closed pores in the night*, as how to determine the force employed by the rump of the peacock in elevating and expanding his splendid tail. We suspect that the author's studies have led him amongst the late class of mathematical physicians, and that he is an admirer of the philosopher who discovered that the celerity of the motion of the excrements through the intestines, was exactly in the inverse ratio of the square of the distances. But to proceed; the author observes that

"A regular and alternate rise and fall of temperature commonly takes place every four and twenty hours, according to the absence and presence of the sun, the fountain of heat; consequently, those who are exposed continually to the temperature of the atmosphere, suffer a diurnal flux and reflux in the vascular system. During the day, the pores of the skin are open; but, during the night, they become closed, when a vascular plethora takes place; that in warm latitudes this change takes place more suddenly than in cool ones, from the more sudden rising and setting of the sun: that in damp situations, where, from the shallowness and opacity of the water, the rays of the sun are obstructed so as to cause rapid evaporation from the surface, the atmosphere, while the sun is up, dissolves a very great quantity of water, with which it becomes saturated; but, the moment the sun goes down, the atmosphere, being no longer able to hold so great a quantity of water in solution, lets go part of it, which we see in the form of fog. The atmosphere now, to use the language of chemistry, becoming a much better conductor of caloric than before, strikes a chilliness into the human frame, shuts up the pores, and stops the flow of perspiration.

perspiration. As the intestinal absorption is not so immediately under the influence of the air, it probably continues, so that the fluids begin to accumulate from this moment. If the person is comfortably lodged, and enjoys repose, the perspiration may be restored during the night, so as to prevent any considerable mischief. Or, even if the person be exposed to the night air, the accumulation may go on till morning, when the returning warmth of the sun may restore the equilibrium, or, at least, restore the fluids to their natural quantity, without the previous accumulation being perceived.

"But, should any accidental circumstance occur in the morning to prevent the return of perspiration; and if, at the same time, the other exciting organs obstinately refuse to transmit the superabundant fluids, the inconvenience is soon perceived. A lassitude and drowsiness come on, to which is soon added an aching pain in the head, back, and limbs, along the course of the large vessels, but usually referred to the bones. These symptoms increase, till, at length, the muscular coats of the vessels are overpowered by the increasing pressure; and it seems to be during this struggle between the vessels and their distending load, that we see those violent convulsions of the frame called shivering.

"The period at which the vascular distention arrives at this pitch, depends, of course, upon the rapidity of the previous accumulation; and, perhaps, in some measure, upon the strength and resistance in the constitution of the patient. The quotidian usually declares itself in the forenoon, when the motion of the body, after a state of rest, probably soon renders the load of fluids insupportable. As, in this form of intermittent, the accumulation is most rapid, so the cold stage is soonest overcome by the increasing fluids; but, as the seat of obstruction is at the surface, and may be more complete than in other forms of the disease, the hot stage is protracted, from the greater difficulty of removing this obstruction.

"In the tertian, the disproportion between the ingress and egress of fluids being less than in the quotidian, a longer period is required before the vascular system is filled so as to produce a fit; and, for the reasons assigned above, the paroxysm is deferred till a later period of the day: and, from the slowness of accumulation, the cold stage is of longer duration.

"In the quartan, so slow is the vascular accumulation, that an interval of three days is required before insupportable plenitude is occasioned. And here again the paroxysm is generally deferred till the afternoon, from the more gradual progress of accumulation. The cold stage is longer than in the two preceding forms of the disease.

"When the vessels arrive at the inconvenient state of plenitude that occasions a paroxysm, sickness and vomiting, the common attendants of vascular plethora, very often occur. I shall notice, here, that these symptoms happen when the menses are suppressed in the first stage of pregnancy, in ideopathic apoplexy, in nephritic complaints complaints that obstruct the flow of urine, &c. When fever is not attended with obstruction in the biliary ducts, the pressure of the fluids accumulated in the liver may create an unusual flow through them, and this may be converted into bile in its passage. It may be again pressed out of the biliary canals by the mechanical effort of vomiting. Nay, in the most violent cases, the blood seems to be pressed through these canals, either mixed with bile, or imperfectly converted into that secretion. Such appears to be the case in black vomit, or that which resembles coffee-grounds."

Dr. Calvert, who appears to be a man of considerable observation, was once inclined to adopt the opinion of many others, that the head was the principal part affected in fever, or that it was the seat of the disease; but subsequent experience has convinced him that this is not the case. To support his last opinion, he states the dissection of two heads, one of a patient who died of an acute remittent fever, the other of a patient who died of phthisis pulmonalis. The bodies being covered, and nothing but the brains exposed, the appearances in each were so similar, that neither the doctor nor his assistants could "say which was the fever patient and which the pulmonic." He thus explains the determination of blood to the head.

"The fulness of the blood-vessels of the brain, as seen on dissection, depends, in a great measure, upon the position in which the patient dies; for, as the blood after death always flows to the most depending part of the body, it will flow to the head, if that lies low; although, on the other hand, as the pressure of the atmosphere is taken off by the structure of the cranium, the blood cannot entirely quit the head so as to leave the vessels flaccid."

We have now stated enough to enable our readers to judge of the merits of this ingenious performance, which offers much food for discussion in a medical society.

---

